# Impact of symptomatic comorbidities on heatstroke outcomes: A retrospective nationwide cohort study

**DOI:** 10.1038/s41598-026-37133-6

**Published:** 2026-01-26

**Authors:** Koichi Inukai, Ryo Narikawa, Suguru Kishitani, Takamasa Takeuchi, Kentaro Takeda, Hiroyuki Kaneko, Syuhei Ikeda, Masahiro Fukuda, Junichiro Kato, Hirotada Kittaka, Yusuke Ito, Hirotaka Sawano

**Affiliations:** 1 Senri Critical Care Medical Center, Saiseikai Senri Hospital, 1-1-6 Tsukumodai, Suita, Osaka 565-0862 Japan; 2 Department of Signal Transduction, Research Institute for Microbial Diseases, The University of Osaka, 3-1 Yamada-oka, Suita, Osaka 565-0871, Japan

**Keywords:** Heatstroke-related mortality, Symptomatic comorbidities, Climate change, Survival analysis, Heat stress, Risk factors, Environmental sciences, Environmental impact

## Abstract

With global temperatures rising due to climate change, heatstroke is emerging as a public health concern, particularly among older adults. The population of Japan is aging, with heatstroke causing frequent hospitalizations and deaths. This study investigated the impact of symptomatic comorbidities, including cardiovascular, pulmonary, and renal disorders, on prognosis of heatstroke. Data were collected from 2,373 adult patients diagnosed with heat-related illnesses during 2017–2021 across multiple centers in Japan. Among them, 608 patients (25.6%) had at least one comorbidity. Patients were compared based on the presence and number of comorbidities. Statistical analyses, including logistic regression and Kaplan–Meier survival curves after propensity-score matching, were performed to evaluate the relationship between comorbidities and in-hospital mortality. The results showed significantly higher in-hospital mortality among patients with comorbidities compared to those without (15.3% vs. 10.9%, p = 0.005). Although mortality was higher in those with multiple comorbidities, the difference was not statistically significant. Respiratory comorbidities emerged as a significant risk factor for heatstroke-related mortality (odds ratio = 2.93, 95% CI = 1.53 – 5.61). Survival analysis further demonstrated a lower survival probability in patients with comorbidities, suggesting that symptomatic comorbidities, particularly respiratory diseases, notably influenced heatstroke outcomes and require focused preventive strategies.

**Supplementary Information:**

The online version contains supplementary material available at 10.1038/s41598-026-37133-6.

## Introduction

As global temperatures continue to rise owing to climate change, extreme summer temperatures, which considerably impact health and ecosystem well-being, have been increasingly reported across various regions worldwide^1,2^. For example, severe heat waves in Europe have significantly increased heat stress-related health issues^3^. Specifically, the 2003 heat wave in France, characterized by peak temperatures of 40 °C in August, resulted in a serious public health crisis and over 14,000 associated deaths, predominantly among older adults^4^. In North America, the extreme heat caused by a meteorological phenomenon known as a ‘heat dome’ in 2021 led to 619 reported deaths in British Columbia, Canada, within one week^5^. The fatalities were particularly concentrated among the elderly, individuals with pre-existing health conditions, and those who were socially isolated.

Heatstroke, a severe manifestation of heat stress, occurs when the thermoregulatory mechanisms of the body fail. This condition is influenced by external temperature, internal heat production, and physiological regulatory systems, such as the circulatory and endocrine systems^6^. Therefore, even under similar thermal conditions, older adults, who generally have a diminished regulatory capacity, are more susceptible to heatstroke than younger and healthier individuals.

Further, France has emphasized the critical need to protect older individuals, who are at higher risk of experiencing heat-related illnesses. Epidemiological studies on heatstroke in the general population are also essential for assessing broader societal risks. Similarly, Japan faces substantial health challenges owing to abnormally high summer temperatures. In 2018, Japan experienced a surge in heat-related illnesses and a corresponding increase in heat-related mortality^7^. In Japan, a nation with one of the highest aging populations globally, an estimated 60% of heatstroke-related hospitalizations and 80% of heatstroke-related deaths occur in older adults^8^. As the population in developed countries continues to age, the incidence of heatstroke and its associated mortality are expected to rise. Older adults often suffer from multiple comorbidities such as hypertension and diabetes mellitus. Thus, investigating how these comorbidities influence the risk of heatstroke in older adults and clarifying how they are affected by heatstroke are important. Understanding these interactions also has important public health implications.

Pre-existing medical conditions contribute to increased mortality and morbidity during heat waves^9–13^. Specifically, individuals with cardiovascular or cerebrovascular diseases, respiratory disorders, neuropsychiatric conditions, diabetes mellitus, or renal diseases are particularly vulnerable to heat stress. However, evidence linking these conditions to an elevated risk of heatstroke is insufficient. Further, although some studies have suggested a connection between certain medical conditions and heatstroke^14–16^, the association between heatstroke and these symptomatic comorbidities requires clarification. Furthermore, these previous studies were limited by small sample sizes or only included data corresponding to specific geographic areas. The relationship between comorbidities and heatstroke, particularly mortality, has not been adequately explored on a larger scale. Additionally, the results of most previous studies are limited by unclear definitions of the severity of pre-existing medical conditions or comorbidities. Therefore, it is necessary to evaluate the prognosis of heatstroke in the context of current active medical conditions using a large sample size and based on well-defined comorbidities rather than on past medical history.

The Japanese Association for Acute Medicine (JAAM) has been collecting data on patients diagnosed with heat-related illnesses, such as heatstroke, since 2006 through a nationwide heat-related illness registry (the HsS: the Heatstroke Study, clinical trial number: not applicable). In the present study, we used JAAM registry data to clarify the impact of symptomatic comorbidities on the prognosis of heatstroke; our findings will facilitate targeted interventions to help at-risk populations in the context of heatstroke.

## Methods

### Study design and settings

A retrospective nationwide cohort study, referred to as the HsS, was conducted in Japan using yearly data collected in July–September from 2017 to 2021 by the JAAM. This national database of the Heatstroke and Hypothermia Surveillance Committee of JAAM was established in 2006. The study protocol was approved by the Teikyo University Ethical Review Board for Medical and Health Research (approval number, 17–021-5; board name, Heatstroke STUDY; approval date, May 21, 2020). The study was approved by the institutional review boards of the different hospitals where the patients were admitted. Informed consent was obtained from all study participants using a form approved by Teikyo University Ethical Review Board for Medical and Health Research. All methods were performed in accordance with the relevant guidelines and regulations, including the Declaration of Helsinki.

**Fig. 1:**
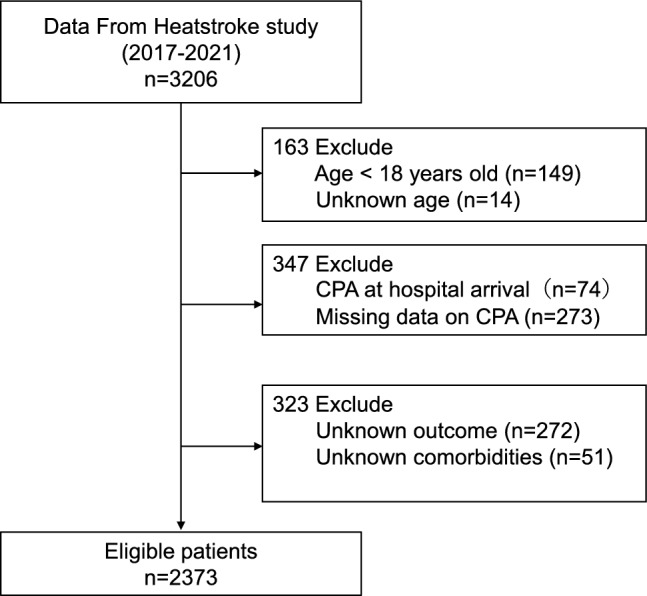
Enrolment of study patients. CPA, cardiopulmonary arrest.

Figure 1 shows the procedure for the inclusion of patients in this study.　According to the JAAM database, 3206 patients were hospitalized for heatstroke between 2017 and 2021. The participating physicians collected information related to hospital admission related to the diagnosis of heat-related illness based on the examination of the medical records of the patients. The collected information was then entered into a web-based data collection system for subsequent analysis.

### Patients

Adult patients (aged ≥ 18 years) who were diagnosed with heat-related illnesses and admitted to the different participating 165 hospitals were studied. The heat-related illnesses were diagnosed using the modified definition of heatstroke (i.e., the mJAAM criteria)^17^ or Bouchama heatstroke criteria^6^ based on the discretion of individual clinicians. Patients who experienced cardiopulmonary arrest upon admission and those with unknown mortality and comorbidities were excluded from this study. In cases of cardiopulmonary arrest at arrival, accurate assessment of admission vital signs and laboratory parameters was not feasible.

### Data collection and definition of symptomatic comorbidities

The primary outcome of this study was in-hospital mortality. The following data were collected for the included patients: age, sex, body mass index (BMI), outdoor onset, comorbidities (liver disease, cardiovascular disease, respiratory disease, immunodeficiency, kidney disease, diabetes mellitus, and psychiatric disease), body temperature, systolic blood pressure, heart rate, respiratory rate, Glasgow Coma Scale (GCS) score (range 3–15, assessing the level of consciousness), cardiopulmonary arrest during prehospital stay, intubation, length of stay in the ICU and hospital, and in-hospital mortality. All vital signs and laboratory parameters were recorded upon arrival at the emergency department. Temperature was recorded as core temperature based on measurements at the rectum, urinary bladder, or esophagus, if available. Otherwise, peripheral temperature based on measurement at the axilla or ear was recorded. Laboratory data, including pH, lactate level (arterial blood gas analysis), sodium, potassium, and chlorine levels, white blood cell and platelet counts, creatine kinase levels, blood urea nitrogen (BUN) and creatinine levels, total bilirubin, aspartate transaminase (AST) and alanine transaminase (ALT) levels, hemoglobin and hematocrit levels, and prothrombin time (PT) activity were measured at the time of admission to the emergency department.

Comorbidities were identified based on the medical records at the time of hospital admission and included those under ongoing treatment at admission. They were regarded as pre-existing conditions rather than complications newly developed after hospitalization. Symptomatic comorbidities included liver disease, defined as cirrhosis, portal hypertension, hepatic failure, and hepatic coma; cardiovascular disease, defined as a state of constant respiratory distress and chest tightness that increased with exertion; respiratory disease, defined as severe mobility impairment due to chronic restrictive or obstructive disease, vascular disease, chronic hypoxemia, hypercarbia, secondary polycythemia, severe pulmonary hypertension (> 40 mm Hg), or being in a ventilator-dependent state; immunodeficiency, defined based on prescriptions of immunosuppressive drugs and long-term or high-dose steroid use, chemotherapy, radiotherapy, leukemia, lymphoma, and AIDS; chronic kidney disease, defined as a condition requiring continuous dialysis; and diabetes mellitus, defined as a condition associated with organ damage. Mental illness was diagnosed by the examining clinicians based on medical records or ongoing psychiatric treatment.

### Statistical analysis

First, the study population was divided into two groups: those with and without comorbidities. Further, the group with comorbidities was stratified into single and multiple comorbidity groups. Then, to examine differences in clinical data between the different groups, including basic characteristics and adjustment variables, continuous variables were compared using the Student’s t-test when normally distributed and the Mann–Whitney U test when non-normally distributed, whereas categorical variables were compared using the chi-square test or Fisher’s exact test, as appropriate. Subsequently, to explore the association between each comorbidity and heatstroke outcome, we performed logistic regression analysis. The outcome variable was in-hospital mortality, and the exposure variables were the comorbidities of interest. The model was adjusted for potential confounders (adjustment variables), including age, sex, BMI, body temperature, and systolic blood pressure upon arrival at the hospital. These adjustment variables were selected considering their potential association with the outcome of interest based on clinical knowledge^18–22^. Additionally, the relationship between the covariates and the outcome of interest was evaluated using odds ratios (OR) and 95% confidence intervals.

Kaplan–Meier curves were generated only for descriptive visualization to illustrate overall survival trends between patients with and without comorbidities. Neither the log-rank test nor the Cox proportional hazards model was used because the proportional hazards assumption was violated—most deaths occurred shortly after admission, resulting in time-dependent hazards and partially crossing survival curves. Therefore, logistic regression was used as the primary analysis to provide valid inference on in-hospital mortality as a binary outcome.

To reduce potential confounding, we adjusted for differences in the above covariates for patients via propensity-score matching using the following algorithm: 1:1 optimal matching with a ± 0.2 caliper and no replacement. We also used standardized difference analysis to measure covariate balance, with an absolute standardized difference above 10% representing a meaningful imbalance.

Sensitivity analyses were additionally performed to assess the robustness of the main findings. In these analyses, we fitted alternative logistic regression models that, in addition to the adjustment variables described above (age, sex, BMI, body temperature, and systolic blood pressure upon arrival at the hospital), also included admission lactate level, platelet count, and log-transformed creatine kinase (CK). These acute-phase biomarkers were considered indicators of acute physiological insult and organ dysfunction at presentation and are likely to lie on the causal pathway between pre-existing comorbidities and in-hospital mortality. Therefore, their inclusion was treated as a sensitivity analysis to avoid overadjustment in the primary models.

Furthermore, an exploratory causal mediation analysis was undertaken to investigate whether acute illness severity at admission mediates the association between respiratory disease and in-hospital mortality. In this framework, pre-existing respiratory disease was specified as the exposure, in-hospital mortality as the outcome, and admission lactate level as the mediator, conceptualized as an indicator of acute physiological insult during the heatstroke episode. Logistic regression was used for the outcome model and linear regression for the mediator model, with adjustment for age, sex, BMI, body temperature, and systolic blood pressure upon arrival at the hospital as baseline confounders. Using the mediation package in R with 1,000 simulations, the average causal mediation effect (ACME), average direct effect (ADE), total effect, and the proportion of the total effect mediated by admission lactate were estimated. In addition, to assess the extent to which a broader set of acute physiological abnormalities could account for this association, a sensitivity mediation analysis was performed using a parallel multiple mediator model. In this model, admission lactate level, platelet count, serum creatinine level, and total GCS score at hospital arrival were simultaneously specified as mediators between pre-existing respiratory disease and in-hospital mortality, while the same baseline confounders (age, sex, BMI, body temperature, and systolic blood pressure) were included as adjustment variables. This multiple mediator analysis was implemented using the *mma* package in R.

In all analyses, statistical tests were two-sided, and a P value < 0.05 was considered statistically significant. All statistical analyses were performed using EZR (Saitama Medical Center, Jichi Medical University, Saitama, Japan, version 1.68), a graphical user interface, or a modified version of R commander (version 2.9–2) (The R Foundation for Statistical Computing, Vienna, Austria, version 4.4.1), which was designed to incorporate statistical functions commonly used in biostatistics.

## Results

### Comparison of patients with and without comorbidities

Among the included patients with heat-related illnesses, 608 had comorbidities (Table 1). Specifically, 140 patients had cardiovascular disease, 69 had respiratory disease, 50 had liver disease, 41 had kidney disease, 74 had diabetes mellitus, 56 had immunodeficiency, and 286 had psychiatric disease. Patients with comorbidities tended to be older (average age, 70.47 years ± 16.34) than those without comorbidities (average age, 67.90 years ± 19.27) (p = 0.003). Additionally, the proportion of males was significantly lower in the group with comorbidities than in that without comorbidities (64.5% vs. 70.8%, p = 0.004). Patients with comorbidities presented with higher body temperatures upon arrival at the hospital (38.66 °C vs. 38.19 °C, p < 0.001) and were more likely to experience heatstroke outdoors (54.2% vs. 47.0%, p = 0.003). They also exhibited higher heart rates (109.68 vs. 104.76 bpm, p < 0.001) and respiratory rates (25.59 vs. 24.51, p = 0.015) than those without comorbidities. However, the two groups showed no significant differences with respect to systolic blood pressure upon arrival at the hospital (p = 0.6). Patients with comorbidities had lower diastolic blood pressures than those without comorbidities (73.95 vs. 76.50 mmHg, p = 0.011).

**Table 1:** Clinical characteristics and outcomes of hospitalized heatstroke patients with and without comorbidities.

Variable	Overall	Without comorbidities	With comorbidities	p-value
n (%)	2373	1765 (74.3)	608 (25.6)	
Male, n (%)	1641 (69.2)	1249 (70.8)	392 (64.5)	0.004
Age	68.56 (18.60)	67.90 (19.27)	70.47 (16.34)	0.003
Outdoor onset, n (%)	1147 (48.9)	822 (47.0)	325 (54.2)	0.003
BMI	22.59 (4.64)	22.57 (4.55)	22.63 (4.89)	0.788
**Vital signs at hospital arrival**				
Body temperature, ℃	38.31 (1.76)	38.19 (1.75)	38.66 (1.77)	< 0.001
Systolic blood pressure, mmHg	126.89 (31.51)	127.09 (31.77)	126.31 (30.76)	0.600
Diastolic blood pressure, mmHg	75.85 (21.11)	76.50 (21.12)	73.95 (20.96)	0.011
Heart rate, bpm	106.02 (28.46)	104.76 (27.95)	109.68 (29.63)	< 0.001
Respiratory rate, /min	24.79 (9.02)	24.51 (8.56)	25.59 (10.21)	0.015
Total GCS	11.76 (4.18)	14.00 [3.00, 15.00]	14.00 [3.00, 15.00]	0.19
**Laboratory Data**				
pH	7.41 (0.14)	7.41 (0.15)	7.42 (0.09)	0.265
Lactate, mmol/l	3.86 (4.47)	3.86 (4.33)	3.85 (4.88)	0.948
Base excess, mmol/l	-2.59 (5.25)	-2.62 (5.12)	-2.52 (5.62)	0.716
White blood cell, /µl	10,073.68 (8958.13)	10,091.18 (8937.86)	10,022.85 (9024.02)	0.872
Hemoglobin, g/dl	13.85 (2.71)	15.40 (38.16)	13.83 (16.14)	0.329
Hematocrit, %	40.63 (7.56)	41.46 (11.50)	38.84 (7.40)	< 0.001
Platelet count, × 10^4^/µl	27.09 (36.51)	28.09 (39.16)	24.24 (27.35)	0.027
BUN, mg/dl	24.4 [17.7, 35.7]	24.45 [18.00, 35.30]	24.10 [17.00, 36.70]	0.529
Creatinine, mg/dl	1.40 [0.97, 2.20]	1.44 [0.96, 2.20]	1.40 [1.00, 2.11]	0.821
Total bilirubin, mg/dl	0.90 [0.70, 1.40]	1.00 [0.70, 1.40]	0.90 [0.60, 1.35]	0.001
ALT, U/l	24 [15, 43]	24 [16, 42]	24 [15, 44]	0.562
AST, U/l	35 [24, 63]	34 [24, 59]	36 [23, 80]	0.102
Creatine kinase, U/l	262 [121, 762]	251 [122, 652.75]	300 [119.5, 1076.5]	0.006
Sodium, mmol/l	139.77 (7.45)	139.90 (7.35)	139.19 (9.37)	0.163
Potassium, mmol/l	4.21 (1.62)	4.54 (10.92)	4.85 (16.37)	0.600
PT activity, %	87 [69, 100]	88 [71, 100]	84 [65, 97]	0.001
**Outcome**				
Ventilator, n (%)	310 (13.6)	222 (13.1)	88 (15.0)	0.235
Length on ventilator, day	0 [0, 2]	0 [0, 1]	0 [0, 2]	0.870
Length of ICU stay, day	0 [0, 3]	0 [0, 3]	0 [0, 3]	0.038
Length of hospital stay, day	5 [2, 13]	5 [2, 12]	7 [3, 14.5]	< 0.001
In-hospital mortality, n (%)	285 (12.0)	192 (10.9)	93 (15.3)	0.005

Data are presented as mean (standard deviation) for continuous variables and number (percentage) for categorical variables. For non-normally distributed variables, values are expressed as median [interquartile range, IQR]. Abbreviations: BMI, Body Mass Index; GCS, Glasgow Coma Scale; BUN, Blood Urea Nitrogen; ALT, Alanine Aminotransferase; AST, Aspartate Aminotransferase; PT, Prothrombin Time; ICU, Intensive Care Unit.

Further, the laboratory data revealed several significant differences between the patients with and without comorbidities. Specifically, hematocrit levels and platelet counts were significantly lower in patients with comorbidities than those without comorbidities (38.84% vs. 41.46%, p < 0.001 and 24.24 vs. 28.09, p = 0.027, respectively). Creatine kinase levels were also significantly higher in patients with comorbidities than in those without comorbidities (300 vs. 251, p = 0.006). Additionally, PT activity was significantly longer in patients with comorbidities than in those without comorbidities (88 vs. 84, p = 0.001).

The number and proportion of deaths stratified by each comorbidity are summarized in Supplementary Table S1. Patients with respiratory disease had the highest mortality rate (26.1%), followed by those with diabetes mellitus (16.2%) and psychiatric disease (15.7%), consistent with the regression analysis results.

Regarding outcomes, in-hospital mortality was significantly higher among patients with comorbidities than among those without comorbidities (15.3% vs. 10.9%, p = 0.005). Furthermore, patients with comorbidities exhibited longer hospital stays (7 vs. 5 days, p < 0.001). However, no significant difference was observed between the two groups with respect to ventilator use (p = 0.235) or duration of ventilator use (p = 0.870).

Taken together, patients with comorbidities hospitalized for heatstroke exhibited more severe clinical presentations, as evidenced by significant differences in vital signs, laboratory markers, and clinical outcomes relative to those without comorbidities. These findings highlight the importance of considering underlying health conditions in the management and prognosis of patients with heatstroke.

### Comparison of patients with single and multiple comorbidities

Among the 608 patients with comorbidities, two groups were identified: those with a single comorbidity and those with multiple comorbidities (Table 2). The proportion of male patients did not differ significantly between the two groups (63.9% vs. 67.8%, p = 0.546); however, patients with multiple comorbidities were significantly older than those with a single comorbidity (74.44 vs. 69.81 years, p = 0.014). No significant differences were observed in the proportion of patients with outdoor heatstroke onset (p = 0.411) and BMI (p = 0.287) between the two groups.

**Table 2:** Clinical characteristics and outcomes of hospitalized heatstroke patients with single and multiple comorbidities.

Variable	Single comorbidity	Multiple comorbidities	p-value
n	521	87	
Male, n (%)	333 (63.9)	59 (67.8)	0.546
Age	69.81 (16.79)	74.44 (12.72)	0.014
Outdoor onset, n (%)	275 (53.4)	50 (58.8)	0.411
BMI	22.73 (4.95)	22.08 (4.52)	0.287
**Vital sign at hospital arrival**			
Body temperature, ℃	38.67 (1.81)	38.60 (1.56)	0.730
Systolic blood pressure, mmHg	125.96 (30.13)	128.41 (34.44)	0.495
Diastolic blood pressure, mmHg	73.77 (20.67)	75.04 (22.76)	0.607
Heart rate, bpm	109.79 (29.91)	108.99 (28.11)	0.815
Respiratory rate, /min	25.61 (10.46)	25.46 (8.67)	0.903
Total GCS	11.55 (4.15)	11.63 (3.93)	0.876
**Laboratory Data**			
pH	7.42 (0.09)	7.41 (0.08)	0.665
Lactate, mmol/l	3.92 (5.17)	3.38 (2.47)	0.385
Base excess, mmol/l	-2.54 (5.68)	-2.44 (5.29)	0.892
White blood cell, /µl	10,107.61 (9293.78)	9517.26 (7233.57)	0.575
Hemoglobin, g/dl	13.29 (2.63)	12.48 (2.51)	0.008
Hematocrit, %	39.15 (7.39)	37.03 (7.24)	0.014
Platelet count, × 10^4^/µl	24.83 (28.46)	20.76 (19.37)	0.199
BUN, mg/dl	24.00 [16.83, 35.35]	27.00 [18.40, 42.70]	0.106
Creatinine, mg/dl	1.30 [0.98, 2.04]	1.72 [1.23, 3.20]	< 0.001
Total bilirubin, mg/dl	0.90 [0.60, 1.30]	1.00 [0.70, 1.50]	0.032
ALT, U/l	24 [15, 44]	22 [14, 38]	0.455
AST, U/l	36 [23, 78]	36 [25, 102]	0.401
Creatine kinase, U/l	303 [121, 1129]	279.5 [119, 974]	0.642
Sodium, mmol/l	139.33 (7.77)	139.83 (7.48)	0.582
Potassium, mmol/l	4.18 (1.02)	4.24 (0.75)	0.603
PT activity, %	85 [65.35, 97.25]	79.9 [60, 96]	0.260
**Comorbidities**			
Cardiovascular disease, n (%)	104 (20.0)	36 (41.4)	< 0.001
Respiratory disease, n (%)	38 (7.3)	31 (35.6)	< 0.001
Liver disease, n (%)	29 (5.6)	21 (24.1)	< 0.001
Kidney disease, n (%)	16 (3.1)	25 (28.7)	< 0.001
Diabetes mellitus, n (%)	45 (8.6)	29 (33.3)	< 0.001
Immunodeficiency, n (%)	40 (7.7)	16 (18.4)	0.004
Psychiatric disease, n (%)	249 (47.8)	37 (42.5)	0.417
**Outcome**			
Ventilator, n (%)	77 (15.3)	11 (13.3)	0.741
Length on ventilator, day	0 [0, 2.]	0 [0, 1.25]	0.813
Length of ICU stay, day	0 [0, 3.]	0 [0, 4]	0.915
Length of hospital stay, day	7 [3, 14.75]	5 [3, 13]	0.201
In-hospital mortality, n (%)	74 (14.2)	19 (21.8)	0.077

Data are presented as mean (standard deviation) for continuous variables and number (percentage) for categorical variables. For non-normally distributed variables, values are expressed as median [interquartile range, IQR]. Abbreviations: BMI, Body Mass Index; GCS, Glasgow Coma Scale; BUN, Blood Urea Nitrogen; ALT, Alanine Aminotransferase; AST, Aspartate Aminotransferase; PT, Prothrombin Time; ICU, Intensive Care Unit.

Further, in terms of vital signs upon arrival at the hospital, body temperature, systolic blood pressure, diastolic blood pressure, heart rate, respiratory rate, and total GCS scores were comparable between the single and multiple comorbidity groups, with no significant differences observed. The laboratory data revealed significantly lower hemoglobin levels (12.48 vs. 13.29 g/dL, p = 0.008) and hematocrit levels (37.03% vs. 39.15%, p = 0.014) in patients with multiple comorbidities than in those with a single comorbidity. Furthermore, patients with multiple comorbidities had higher serum creatinine levels (2.70 vs. 1.81 mg/dL, p < 0.001) and higher total bilirubin levels (1.0 vs. 0.9 mg/dL, p = 0.032) than those with a single comorbidity. Other laboratory parameters, such as white blood cell count, lactate levels, and creatine kinase levels, did not differ significantly between the two groups.

In terms of clinical outcomes, ventilator use, number of days of ventilator use, and length of ICU stay were not significantly different between the two groups. In-hospital mortality was higher for patients with multiple comorbidities than for those with a single comorbidity. However, the difference was not statistically significant (21.8% vs. 14.2%, p = 0.077). The hospital stay duration was also comparable between the two groups (p = 0.201). Taken together, patients with multiple comorbidities tended to have higher mortality rates, although the difference was not statistically significant.

### Effects of comorbidities on heatstroke outcomes

Table 3 summarizes both unadjusted and adjusted odds ratios for each comorbidity. In the unadjusted model, respiratory comorbidities and psychiatric disorders were associated with in-hospital mortality.

**Table 3:** Multivariable Analysis of the Association Between Comorbidities and In-Hospital Mortality in Heatstroke Patients.

Comorbidity	Unadjusted OR (95% CI)	Adjusted OR (95% CI)	p-value (Adjusted)
Respiratory disease	2.66 (1.51 – 4.68)	2.93 (1.53 – 5.61)	0.001
Psychiatric disease	1.42 (1.00 – 2.01)	1.52 (0.99 – 2.31)	0.051
Cardiovascular disease	1.19 (0.72 – 1.96)	1.05 (0.58 – 1.86)	0.875
Diabetes mellitus	1.49 (0.77 – 2.91)	1.41 (0.64 – 3.09)	0.389
Liver disease	1.40 (0.66 – 2.98)	1.48 (0.64 – 3.35)	0.353
Kidney disease	0.70 (0.25 – 1.96)	0.70 (0.23 – 2.11)	0.534
Immunodeficiency	0.60 (0.23 – 1.55)	0.40 (0.13 – 1.18)	0.097

OR, Odds Ratio; CI, Confidence Interval.

After adjusting for adjustment variables (age, sex, BMI, body temperature, and systolic blood pressure), only respiratory comorbidities remained significantly associated with mortality (adjusted OR = 2.93, 95% CI: 1.53–5.61, p = 0.001).

Other comorbidities—including psychiatric disease (OR: 1.52, 95% CI: 0.99–2.31, p = 0.051), cardiovascular disease (OR: 1.05, 95% CI: 0.58–1.86, p = 0.875), diabetes mellitus (OR: 1.41, 95% CI: 0.64–3.09, p = 0.389), and liver disease (OR: 1.48, 95% CI: 0.64–3.35, p = 0.534)—did not show statistically significant associations with in-hospital mortality. Kidney disease (OR: 0.70, 95% CI: 0.23–2.11, p = 0.534) and Immunodeficiency (OR: 0.40, 95% CI: 0.13–1.18, p = 0.097) also showed no significant association, though a trend toward a protective effect was observed. In sensitivity analyses incorporating additional laboratory covariates, the association between respiratory comorbidities and in-hospital mortality remained materially unchanged (Supplementary Table S2). These results suggest that respiratory comorbidities may be critical risk factors affecting mortality in patients with heatstroke; however, the other conditions may have limited effects.

In addition, when the number of comorbidities was treated as a continuous variable in the multivariable logistic regression model, a higher comorbidity burden was significantly associated with increased in-hospital mortality (adjusted OR = 1.32, 95% CI: 1.10–1.59).

In an exploratory causal mediation analysis using admission lactate level as a mediator of the association between respiratory disease and in-hospital mortality, respiratory disease remained strongly associated with an increased risk of death, whereas the mediated component via lactate was negligible. The total effect of respiratory disease on in-hospital mortality was 0.17 (95% CI 0.05–0.30, p = 0.002), but the average causal mediation effect through admission lactate was very small and not statistically significant (ACME 0.0007, 95% CI -0.012 to 0.014, p = 0.876). The estimated proportion of the total effect mediated by lactate was only 0.3% (95% CI -10.0 to 9.6%, p = 0.878) (Supplementary Table S3). In a subsequent parallel multiple mediator model that simultaneously included admission lactate level, platelet count, serum creatinine level, and total GCS score as mediators, the combined indirect effect through these four markers remained similarly minimal and non-significant, and the direct effect of respiratory disease on in-hospital mortality was essentially unchanged (Supplementary Table S4). Taken together, these findings indicate that the excess mortality risk associated with respiratory disease in patients with heatstroke is not materially explained by acute illness severity at admission as captured by standard biochemical and neurological markers, and is instead largely attributable to mechanisms that are not captured by these indices.

### Survival probabilities of patients after hospitalization

**Fig. 2:**
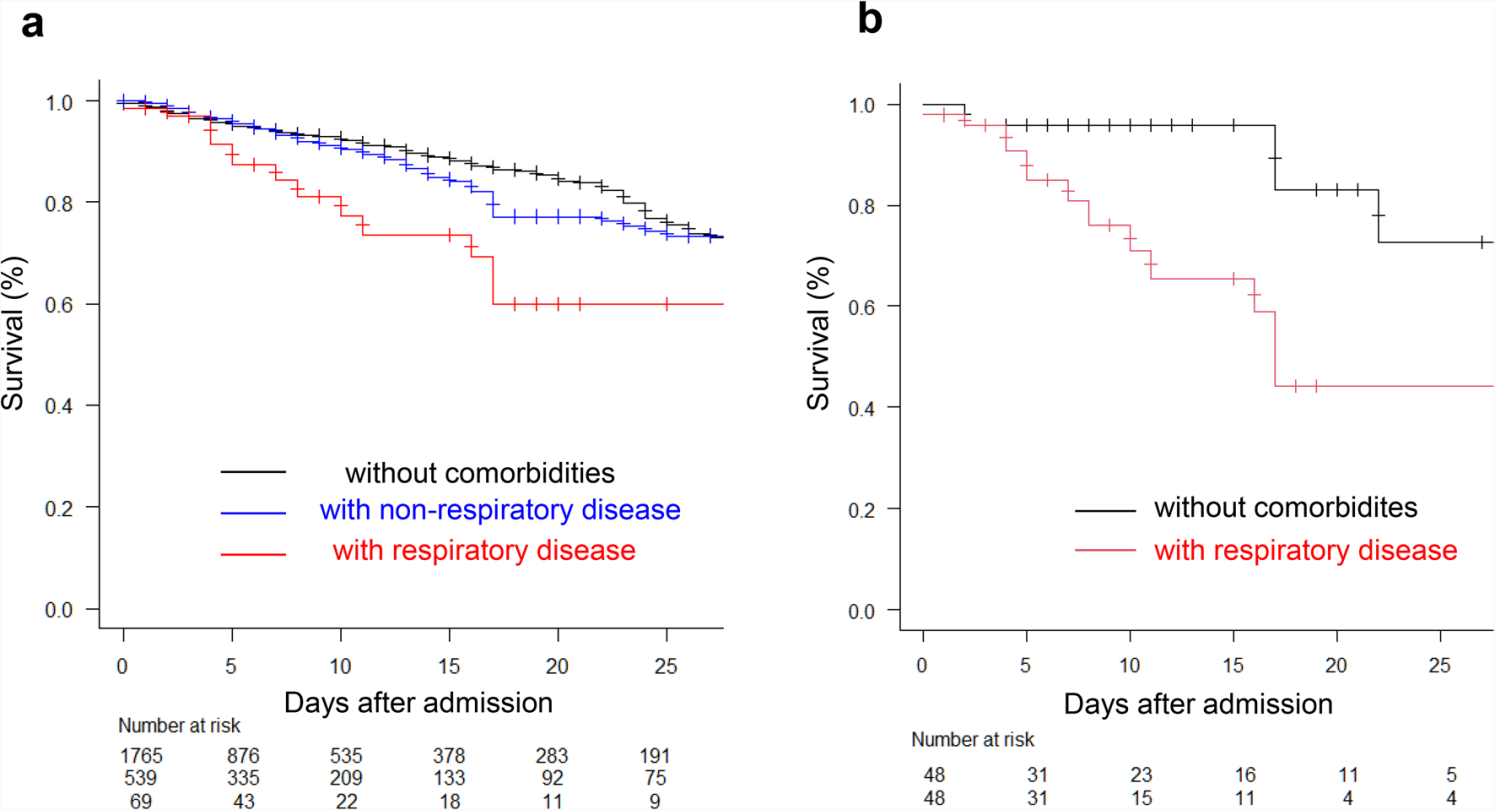
Kaplan–Meier survival curves for patients hospitalized with heatstroke. (**a**) Survival probabilities among three mutually exclusive groups: patients without any comorbidities, patients with non-respiratory comorbidities, and patients with respiratory comorbidities. (**b**) Kaplan–Meier survival curves after propensity-score matching (PSM), comparing patients with respiratory comorbidities and those without any comorbidities under balanced baseline characteristics. The number of patients at risk at each time point is shown at the bottom.

Two Kaplan–Meier curves (Fig. 2) were generated to illustrate the survival probabilities of the included patients after hospitalization, focusing on comorbidity status.

Figure 2a shows survival curves for three mutually exclusive groups: patients without comorbidities, those with non-respiratory comorbidities, and those with respiratory comorbidities. This classification enabled a straightforward comparison of overall survival patterns across clinically distinct categories. Patients with respiratory comorbidities exhibited the lowest survival throughout the 25-day observation period, with survival decreasing to approximately 60% by day 25. Patients with non-respiratory comorbidities showed intermediate survival, and those without comorbidities maintained the highest survival probability, remaining above 75% at day 25. Although this analysis was descriptive and not formally tested, the consistent trend suggests that respiratory comorbidities are associated with a particularly poor prognosis in hospitalized patients with heatstroke.

Figure 2b presents the Kaplan–Meier survival curves after propensity score matching (PSM), comparing patients with respiratory comorbidities and those without any comorbidities under balanced baseline characteristics (Supplementary Table S5).

Even after matching, patients with respiratory comorbidities showed a persistently lower survival probability during hospitalization, and these results collectively indicate that respiratory comorbidities are the most important determinant of poor prognosis among hospitalized patients with heatstroke, consistently demonstrated in both unadjusted and PSM analyses.

## Discussion

In this nationwide registry study, we found that among various symptomatic comorbidities, only respiratory diseases were significantly associated with in-hospital mortality after heatstroke. This finding highlights the potential vulnerability of patients with pre-existing respiratory dysfunction to heat-induced systemic injury.

A 2004 study in the UK showed a significant 5.44% (95% CI: 1.92–9.09) increase in hospital admissions due to respiratory disease for every 1 °C increase in mean daily temperature using 23 °C as a threshold. Further, a 10.86% increase in hospital admission rate (95% CI: 4.44–17.67) for patients aged ≥ 75 years was reported^10^. Reviews in the UK and reports from China have also shown an increase in heat-related deaths at temperatures above thresholds, with respiratory disease-related deaths showing greater susceptibility to heat than cardiovascular disease-related deaths^23,24^.

Similarly, a higher relative risk of death during heat waves has been reported for subjects with a history of hospitalization due to chronic respiratory disease^12,25–27^. Exposure to extreme heat may also induce dyspnea and cardiovascular effects in patients with chronic obstructive pulmonary disease^28^. Additionally, heat-induced physiological changes, such as dehydration, may be a risk factor for pulmonary dysfunction^29^. Collectively, previous studies indicate that chronic respiratory diseases such as COPD and interstitial pneumonia are associated with impaired thermoregulatory capacity and increased mortality risk during heat exposure; however, direct evidence elucidating the physiological pathways by which pulmonary dysfunction affects thermoregulation remains limited^30^. Under heat stress, impaired gas exchange, reduced respiratory evaporative heat loss, and amplified systemic inflammatory responses may interact, but the relative contributions and causal mechanisms are not yet well understood. Therefore, further studies are warranted to elucidate how different types of respiratory diseases influence thermoregulatory responses under heat stress.

Our exploratory causal mediation analyses also provided additional insight into the pathways linking respiratory disease to heatstroke-related mortality. Although respiratory disease was clearly associated with an increased risk of in-hospital death, the proportion of this excess risk mediated by admission lactate—used as a representative marker of acute physiological insult—was very small. The fraction of the effect explained by lactate alone was close to zero and not statistically significant. Furthermore, in a parallel multiple mediator model that simultaneously included admission lactate level, platelet count, serum creatinine level, and total GCS score as mediators, the combined indirect effect through these four markers remained similarly minimal and non-significant, and the direct effect of respiratory disease on in-hospital mortality was essentially unchanged. Taken together, these findings indicate that the increased mortality risk associated with respiratory disease in patients with heatstroke cannot be fundamentally explained by acute illness severity at admission as captured by standard biochemical and neurological markers, and instead support the notion that chronic respiratory impairment and reduced cardiopulmonary reserve may play a more central role in limiting the ability of these patients to tolerate and recover from heat-induced organ dysfunction.

In contrast, other comorbidities did not show statistically significant associations with in-hospital mortality. This lack of association may reflect multiple interacting factors, including limited statistical power, heterogeneity in disease severity, and competing risks among elderly patients. These factors may have attenuated the apparent effects of other comorbidities, and the results should be interpreted cautiously rather than concluding that no effect exists.

Several studies have reported the association between pre-existing mental illness and heatstroke. Patients with mental illness, such as schizophrenia and dementia, are at higher risk of heatstroke-related mortality^13,15,27,28^ owing to reduced cognitive environmental awareness, reduced adaptive behavioral dynamics, such as wearing appropriate clothing and fluid intake, reduced ability to manage the environment, and the side effects of medications, such as antipsychotics, selective serotonin reuptake inhibitors, and antidepressants^15,28^. Moreover, hot environments exacerbate mental illness. A meta-analysis of 53 studies focusing on the association between heat exposure and mental health-related mortality and morbidity^31^ showed that for every 1 °C increase in temperature, mental health-related mortality and morbidity increased with relative risks (RR) of 1.022 (95% CI: 1.015–1.029) and 1.009 (95% CI :1.007–1.015), respectively. These findings further confirm the existence of a close relationship between hot environments and mental illness.

Extreme weather and rising temperatures may increase diabetes-related morbidity and mortality given that fluid loss and electrolyte disturbances owing to heat stress may exacerbate poor glucose control^32^. A 6% [OR = 1.06; 95% CI: 1.04, 1.07] increase in diabetes-related hospital admissions for every 5 °C increase in average daily temperature was reported in Brazil^33^, and a 117% increase in diabetes-related mortality during a heat wave was reported in New York City^34^.

Peripheral neuropathy owing to diabetes may increase the threshold for thermoregulation, thereby causing individuals to become insensitive to changes in ambient temperature. Further, the drugs required for treatment may cause dehydration, reduce cutaneous blood flow, and decrease sweating, possibly resulting in impaired thermoregulation^35,36^. In exertional heatstroke, liver injury is common and is typically characterized by moderate increases in AST levels. Liver injury is generally reversible and asymptomatic; however, it is occasionally accompanied by severe injury or acute liver failure, both of which have poor prognoses^37^.

Heat stress triggers thermoregulatory responses, causing splenic vasoconstriction and cutaneous vasodilation, diverting blood from organs to the skin surface so that it can dissipate heat into the environment. This decrease in splenic blood flow leads to hypoperfusion and ischemia in intestinal and hepatic cells, resulting in the production of reactive oxygen and nitrogen species, which promote mucosal injury and increase intestinal permeability. Further, under such conditions, endotoxins can leak into systemic circulation, causing thermoregulatory failure, circulatory shock, and heatstroke^38–41^.

Additionally, animal models exposed to environmental heat stress show increased radical portal vein content, suggesting that hyperthermia induces cellular hypoxic stress in the liver and intestine^39^. However, reports on whether liver disease increases the risk of heatstroke are limited. Regardless, it is likely that liver diseases have a significant effect on heatstroke outcomes for several reasons. First, liver diseases reduce the detoxification capability of the liver, and during heatstroke, many metabolic by-products and inflammatory substances are produced. If liver detoxification function is impaired, these substances can accumulate in the liver, and thus, worsen the condition. Second, the liver is involved in energy metabolism, including glycogen storage and gluconeogenesis. Under liver disease conditions, these functions are disrupted, leading to insufficient energy supply during heatstroke. Therefore, patients experience amplified fatigue and weakness. Third, the liver plays a key role in blood coagulation owing to its ability to produce clotting factors. Liver diseases can disrupt blood coagulation balance, and the dehydration and circulatory disturbances associated with heatstroke can exacerbate this issue, increasing the severity of heatstroke symptoms.

Several studies have reported an increased risk of heatstroke-related hospitalization or death for patients with symptomatic cardiovascular diseases^30,35,42,38^. However, contrary to our findings, a study by Schifano et al.^28^ reported a lower risk of death on the day of the heat wave for hospitalized patients with cardiovascular diseases; however, this difference was not statistically significant. Moreover, a lower risk of death from heatstroke was reported for patients who had been hospitalized for ischemic disease. In the present study, we used a strict definition of symptomatic comorbidities for patients with cardiovascular disease. Given that these cardiovascular symptoms are exacerbated by exertion, these symptoms may not have been exacerbated by hospitalization owing to heatstroke because the patients were at rest. Therefore, cardiovascular symptoms showed no significant association with the risk of death but may be a risk factor for hospitalization^35^. Semenza et al.^35^ reported an increase in the number of chronic cardiovascular disease diagnoses during heat waves, suggesting that vulnerable individuals cannot implement appropriate cardiac compensation measures, such as the increased cardiac output required during heat stress.

Increased^35^ and decreased^28^ mortality risks owing to heat stress for patients with renal diseases have been reported. Under heat exposure and physical exertion, blood is redistributed from the splenic and renal vascular beds. Older individuals, with reduced left ventricular function cannot overcompensate for this physiological phenomenon, and the resulting decrease in ventricular function may reduce the perfusion of the kidneys and liver^35^. Reports on the relationship between immune disorders and heatstroke are limited. However, secondary generalized anhidrosis, a common sweating disorder and a risk factor for heatstroke, has been attributed to abnormalities in the functioning of eccrine sweat glands, sympathetic abnormalities, autoimmune diseases, and medications. The involvement of immune disorders has also been presumed. Although immunodeficiency appeared to show a protective trend in our analysis, this association was not statistically significant. The small number of patients with immunodeficiency and heterogeneity in treatment strategies, such as ongoing immunosuppressive therapy or regular medical follow-up, may have influenced outcomes. It is also biologically plausible that mild or controlled immunosuppression could attenuate the hyperinflammatory and cytokine-mediated responses characteristic of severe heatstroke. Therefore, this finding should be interpreted cautiously, as it may reflect selection bias or therapeutic modulation rather than a true protective effect of immunodeficiency.

A strength of this study, compared to previous reports that were limited to symptomatic and active comorbid conditions, was the use of a large sample size to examine the risk of death during heatstroke-related hospitalization. This larger sample size allowed for a more detailed examination of health deterioration during high-temperature periods. Moreover, our analysis was based on well-defined comorbidities with propensity-score matching. Further, multivariate analysis, which adjusted for covariates, showed that concomitant diseases, with the exception of respiratory diseases, had no significant effect on heatstroke-related in-hospital mortality. Given that the covariates were likely adjusted for the severity of heatstroke to some extent, the exacerbation of comorbidities and complications due to heat stress may have played a role in reducing mortality. However, accurately determining whether the observed deaths resulted from worsening comorbidities or multi-organ failure associated with heatstroke is challenging because it was not possible to ascertain the cause of death. Accessibility to hemodialysis, including dialysis treatment, in Japan may also influence the risk of death during heatstroke hospitalization in patients with liver or renal diseases.

## Limitations

Our study has a few limitations. This was a retrospective nationwide cohort study based on data collected from medical records, which could have introduced several forms of bias, including selection and information biases, thereby limiting the ability to establish causal relationships. Moreover, as the study was conducted in Japan, the generalizability of the findings may not be feasible for regions with different climates, healthcare systems, or demographic characteristics. Although all data were collected using a standardized registry format, variability in the timing and methods of measurement across institutions may have led to potential measurement or reporting bias. Additionally, the definitions used for comorbidities may not accurately reflect the severity or specific types of these conditions, possibly leading to classification bias. Furthermore, clinical and laboratory data, such as body temperature, were obtained using different methods across medical institutions, leading to possible measurement and reporting bias. In particular, the ambiguous definition of mental illness may have allowed variability in clinical judgment among physicians, resulting in potential misclassification bias.

Finally, the number of patients at risk became very small in the later follow-up periods, which may reduce the reliability of the Kaplan–Meier survival estimates.

## Conclusions

In summary, the results of the present study revealed that some comorbidities during high temperatures were associated with an increased risk of mortality related to heatstroke. Moreover, multiple comorbidities were associated with an increased risk of heatstroke-related morbidity and mortality, necessitating adequate preventive measures and treatments for vulnerable populations. Further, the frequency of heatstroke is expected to increase in the future as temperatures continue to increase, and health management in the context of comorbidities and pre-existing conditions will likely become an important public health issue. Therefore, a deeper understanding of the relationship between comorbidities and heatstroke is necessary to establish effective intervention measures at the healthcare and community levels. These insights will protect the health of individuals, as well as contribute to improving the health of society overall. Future research should examine indicators, such as the cause of death and worsening of the symptoms of comorbidities, to gain a comprehensive understanding of heat stress and its reciprocal effects on comorbidities.

## Supplementary Information

Supplementary Information.

## Data Availability

The datasets used and/or analyzed during the current study available from the corresponding author on reasonable request.
