# Supplementary material for: Impact of symptomatic comorbidities on heatstroke outcomes: A retrospective nationwide cohort study
Source: Sci Rep. 2026 Jan 26;16:6244. doi: 10.1038/s41598-026-37133-6 (PMC12905226; doi:10.1038/s41598-026-37133-6)
Supplement: Supplementary file 1 — Supplementary Information. [file 41598_2026_37133_MOESM1_ESM.pdf]

Impact of symptomatic comorbidities on heatstroke outcomes: a retrospective nationwide cohort study

Koichi Inukai[1*] PhD, Ryo Narikawa[1] MD, Suguru Kishitani[1] MD, Takamasa Takeuchi[1] MD, Kentaro Takeda[1] MD, Hiroyuki Kaneko[1] MD, Syuhei Ikeda[1] MD, Masahiro Fukuda[1] MD, Junichiro Kato[1] MD, Hirotada Kittaka[1] MD, Yusuke Ito[1] MD, Hirotaka Sawano[1] PhD.

[1]Senri Critical Care Medical Center, Saiseikai Senri Hospital

1-1-6 Tsukumodai, Suita, Osaka 565-0862, Japan

Tel: +81-6-6871-0121

Fax: +81-6-6871-0130

[*]**Corresponding author:** Koichi Inukai, M.D., Ph.D.

Supplementary Table S1: In-hospital mortality by comorbidity among patients with heatstroke

Comorbidity	Total (n)	Deaths (n)	Mortality (%)
Respiratory disease	69	18	26.1%
Psychiatric disease	286	45	15.7%
Cardiovascular disease	140	20	14.3%
Diabetes mellitus	74	12	16.2%
Liver disease	50	9	18.0%
Kidney disease	41	5	12.20%
Immunodeficiency	56	5	8.90%
No comorbidity	1765	192	10.9%

Supplementary Table S2. Sensitivity analysis including lactate, platelet count, and log-transformed creatine kinase (CK) as additional adjustment variables in the logistic regression model.

Comorbidity	Odds Ratio	95% CI	p-value
Respiratory disease	3.76	1.84-7.71	< 0.001
Psychiatric disease	1.23	0.772-1.96	0.384
Cardiovascular disease	1.15	0.615-2.15	0.660
Diabetes mellitus	1.37	0.608-3.08	0.449
Liver disease	1.02	0.395-2.62	0.971
Kidney disease	0.826	0.248-2.75	0.756
Immunodeficiency	0.297	0.082-1.06	0.061

CI, Confidence Interval

Supplementary Table S3. Causal mediation analysis of respiratory disease and in-hospital mortality using admission lactate as mediator.

Effect type	Estimate	95% CI lower	95% CI upper	p-value
ACME (average)	0.0007	-0.012	0.0139	0.876
ADE (average)	0.1687	0.053	0.2985	0.002
Total effect	0.1694	0.054	0.2991	0.002
Proportion mediated (average)	0.0027	-0.1	0.096	0.878

ACME, average causal mediation effect; ADE, average direct effect; CI, confidence interval. Pre-existing respiratory disease was specified as the exposure, in-hospital mortality as the outcome, and admission lactate level as the mediator. Estimates were obtained using the mediation package in R with 1,000 simulations, adjusting for age, sex, body mass index, body temperature, and systolic blood pressure upon arrival at the hospital.

Supplementary Table S4. Parallel multiple mediator analysis of the association between pre-existing respiratory disease and in-hospital mortality

Effect type	Estimate (log-odds)	95% CI lower	95% CI upper	p-value
Total effect	1.3	0.17	1.92	0.001[a]
Direct effect	1.33	0.22	1.94	0.001[a]
Total indirect effect (all mediators)	-0.04	-0.3	0.21	0.880[b]
Indirect effect via lactate	0	-0.09	0.08	0.892[b]
Indirect effect via platelet count	0.03	0	0.14	0.044[b]
Indirect effect via serum creatinine	-0.01	-0.03	0.02	0.152[b]
Indirect effect via total GCS score	-0.06	-0.31	0.12	0.728[b]

Abbreviations: GCS, Glasgow Coma Scale; CI, confidence interval. Estimates are shown on the log-odds scale. “Total effect” denotes the overall effect of pre-existing respiratory disease on in-hospital mortality. “Direct effect” denotes the effect not operating through the specified mediators. “Total indirect effect (all mediators)” represents the combined indirect effect through admission lactate level, platelet count, serum creatinine level, and total GCS score. Effects were estimated using a parallel multiple mediator model implemented with the mma package in R, adjusting for age, sex, body mass index, body temperature, and systolic

blood pressure at hospital arrival.

[a] p-values based on normal approximation (p_norm).

[b] p-values based on bootstrap quantiles (p_quan, 1,000 resamples).

Supplementary Table S5: Baseline characteristics of the matched cohort

Variable	Without comorbidities	Respiratory disease	SMD
n	48	48	
Male, n (%)	31 (64.6)	32 (66.7)	0.044
Age	75.56 (14.32)	75.42 (13.86)	0.010
Outdoor onset, n (%)	24 (50.0)	24 (52.2)	0.043
BMI	22.39 (4.14)	22.62 (6.69)	0.040
**Vital sign at hospital arrival**			
Body temperature, ℃	38.51 (1.68)	38.42 (1.51)	0.057
SBP, mmHg	123.46 (35.89)	126.19 (34.01)	0.078
DBP, mmHg	74.60 (20.91)	72.75 (22.41)	0.086
Heart rate, bpm	105.71 (30.15)	113.58 (27.49)	0.273
Respiratory rate, /min	26.31 (7.08)	26.85 (8.58)	0.069
Total GCS	14 [8.5, 15]	14 [8.25, 14]	0.003
**Laboratory Data**			
pH	7.41 (0.10)	7.40 (0.07)	0.114
Lactate, mmol/l	3.79 (3.35)	3.61 (4.58)	0.044
Base excess, mmol/l	-2.67 (4.12)	-1.78 (6.48)	0.162
White blood cell, /uL	9660.33 (5620.72)	9923.85 (6731.34)	0.042
Hemoglobin, g/dl	13.21 (2.91)	13.28 (2.32)	0.025
Hematocrit, %	38.92 (7.84)	39.64 (6.60)	0.098
Platelet count, x10^4/μl	25.69 (33.30)	21.30 (8.45)	0.181
BUN, mg/dl	24.80 [21.22, 40.62]	23.00 [16.08, 28.05]	0.370
Creatinine, mg/dl	1.39 [1.02, 2.10]	1.31 [1.03, 1.82]	0.191
Total bilirubin, mg/dl	0.90 [0.60, 1.40]	0.82 [0.60, 1.10]	0.428
ALT, U/l	19.5 [13.00, 37.25]	19 [12.75, 39.25]	0.036
AST, U/l	30.5 [21.75, 69.25]	33 [21.75, 81.75]	0.013
Creatine kinase, U/l	178.5 [111, 1080]	210.5 [101.25, 796.5]	0.052
Sodium, mmol/l	141.23 (7.61)	139.79 (6.48)	0.203
Potassium, mmol/l	4.05 (0.62)	4.15 (0.65)	0.155
PT activity, %	82 [65.25, 99]	84.25 [67.5, 97.75]	0.104

Data are presented as mean (standard deviation) for continuous variables and number (percentage) for categorical variables. For non-normally distributed variables, values are expressed as median [interquartile range, IQR]. Propensity score matching (PSM) was performed using age, sex, body mass index (BMI), body temperature, systolic blood pressure (SBP), and lactate level as matching variables. Standardized mean difference (SMD) values <0.1 indicate adequate balance between the groups.

Abbreviations: BMI, body mass index; GCS, Glasgow Coma Scale; BUN, blood urea nitrogen; ALT, alanine aminotransferase; AST, aspartate aminotransferase; PT, prothrombin time.
